# Network pharmacology mechanism of Scutellarin to inhibit RGC pyroptosis in diabetic retinopathy

**DOI:** 10.1038/s41598-023-33665-3

**Published:** 2023-04-20

**Authors:** Na Li, Xi-Liang Guo, Min Xu, Ji-Lin Chen, Yu-Fei Wang, Jie-Sun, Yu-Gao Xiao, An-Shun Gao, Lan-Chun Zhang, Xue-Zheng Liu, Ting-Hua Wang

**Affiliations:** 1 Department of Anatomy, College of Basic Medicine, Jinzhou Medical University, Jinzhou, 121001 China; 2 Animal Center, Kunming Medical University, Kunming, 650500 China; 3 Institute of Neuroscience, Kunming Medical University, Kunming, 650500 China; 4 The First People’s Hospital of Luquan Yi and Miao Autonomous County, Luquan, 651500 China

**Keywords:** Drug discovery, Molecular biology, Neurology, Pathogenesis

## Abstract

To investigate the effect of scutellarin (SCU) in diabetic retinopathy (DR) and explore the associated molecular network mechanism. The animal model of DR was established from diabetic mellitus (DM) rats by intraperitoneally injected streptozotocin (STZ) at dosage 55 mg/kg. Meanwhile, SCU was intraperitoneally administrated to protect retina from cell pyroptosis induced by DM, and cell pyroptosis was detected by using HE, Nissl staining, and immunofluorescence recognition. Moreover, the hub gene involving in pyroptosis in DR was screened by bioinformatics and network pharmacology, designated as Venny intersection screen, GO and KEGG analysis, PPI protein interaction, and molecular docking. Lastly, the expressional change of hub genes were validated with experimental detection. Cell pyroptosis of the DR, specifically in retina ganglion cells (RGC), was induced in DM rats; SCU administration results in significant inhibition in the cell pyroptosis in DR. Mechanically, 4084 genes related to DR were screened from GeneCards and OMIM databases, and 120 SCU therapeutic targets were obtained, by using GeneCards, TCMSP with Swiss Target Prediction databases. Moreover, 357 targets related to pyroptosis were found using GenenCards database, and Drug, disease and phenotypic targets were analyzed online using the Draw Venn Diagram website, and 12 cross targets were obtained. Through GO function and KEGG pathway enrichment analysis, 659 BP related items, 7 CC related items, 30 MF related items, and 70 signal pathways were screened out; Of these, eleven proteins screened from cross-target PPI network were subsequently docked with the SCU, and their expressions including caspase-1, IL-1β, IL-18, GSDMD and NLRP3 in RGC indicated by immunofluorescence, and the mRNA expression for caspase-1 in DR indicated by quantitative PCR, were successfully validated. SCU can effectively protect RGC pyroptosis in DR, and underlying mechanisms are involved in the inhibition of caspase-1, GSDMD, NLRP3, IL-1β and IL-18. Our findings therefore provide crucial evidence to support the clinic practice of SCU for the treatment of DR, and explained the underlying molecular network mechanism.

## Introduction

Diabetic retinopathy (DR), as a common microvascular complication^1^, is usually characterized by specific changes including microaneurysms, beaded veins, and abnormalities of the microvessels in the retina^2^. Of these, total retinal photocoagulation, laser therapy and vitrectomy can reduce vision loss, and there is still no effective prevention and treatment measures for DR up to now. As the incidence of vision loss is still very high in DR, the effective treatment is needing to be developed urgently.

With the development of Chinese medicine, more and more Traditional Chinese medicine has been used for the early treatment of DR. For example, compound Xue shuan tong^3,4^, Ligustrazine Hydrochloride^5^, and Qi Ming Ke Li^6^, have been used to prevent diabetes, but their effect are limited for the treatment of DR, so it is urgent to develop new effective drugs in the treatment of DR. Luckily, scutellarin (SCU), as a flavonoid extracted from the traditional Chinese medicine, exhibits effective anti-inflammatory, antioxidant, anti-fibrosis, anti-tumor, and ameliorating cardio-cerebral ischemia. It is therefore considered as a potential candidate that is possibility to be used for the treatment of DR.

Cell pyroptosis, named as cellular inflammatory necrosis, has been known as a new programmed cell death that is different from cell apoptosis. When pyrotosis occurred, cells swell constantly up to cell membrane rupture, resulting in the release of cell contents to activate inflammatory responses. In molecular level, cell pyroptosis is believed to induce the death of monocytes by caspase-1, then caspase-1 activated GSDMD to cleave cell membrane^7^. Of these, activation of NLRP3 inflammasome can promote the maturation and secretion of inflammatory cytokines IL-18 and IL-1β. It has been reported that inhibition of pyroptosis-mediated cell death can significantly inhibit the progression of DR^8^, but whether or not SCU is useful for this process and associated molecular network mechanisms are largely unknown.

Here, we investigated the effect of Scutellarin against pyroptosis in DR and explore underlying molecular network mechanism. HE staining, Nissl staining and immunofluorescence detection were firstly used to observe retinopathy in diabetic animal model. Then, by using network pharmacology method, combined experimental validation, we determined the effect of SCU and explained underlying molecular network mechanism in DR rat.

## Methods

### Animals groups

Adult male Sprague Dawley (SD) rats were purchased from the Department of Experimental Animal center, Kunming Medical University and divided into normal group (n = 10), diabetic mellitus (DM) to induce diabetic retinopathy (DR) group (n = 10) and SCU treatment group (n = 10).

### Model establishment

Rats are fasted for 12 h before modeling. DM rats were induced by intraperitoneally injected streptozotocin (STZ) (Shanghai Yuanye Bio-Technology Co., Ltd., China) at dosage 55 mg/kg, only one times till model was successfully induced. Blood glucose was measured to evaluate whether the DM modeling was successful when the blood glucose was ≥ 16.7 mmol/L at 3 days. This was followed by a last observation at one month until DR was induced, by using routine Optical Coherence tomography (OCT) detection. Lastly, 26 rats consisting 10 normal rats, 8 rats with DR, and 8 rats with DR subjected to SCU administration were used in later experiment.

### SCU administration

Rats were treated with intraperitoneal injection SCU (Kunming Longjin Bio-Technology Co., Ltd., China) with dosage 50 mg/kg, for one-time daily injection from 1 month till 3 months; while the DM group was given the same amount of normal saline administrated. As to the safety of drug, it has been verified in other report^9^.

### Tissue harvest

Three months after administration, 3 rats were anesthetized with 200 mg/kg sodium pentobarbital, then eyeballs were taken and fixed with 4% paraformaldehyde, and other animals were sacrificed freshly to perform q-PCR experiment. Animal care and all operations were performed in accordance with the guidelines and approved by the Ethics Committee of Kunming Medical University, with the approval number is kummu20220894.

### Frozen section

The fixed eyeballs were frozen in 10%, 20% and 30% sucrose solution (4 °C). Optimal cutting temperature compound (OCT) embedding agent (manufacturer SAKURA USA, batch number: 4583) was dropped to cover the tissue and frozen for 30 min. Lastly, Coronal sections were made with a thickness of 10 μm.

### HE staining

The slides were rewarmed at 37 °C for 10 min and washed twice with double steaming water for 1 min each time. The slides were stained with hematoxylin solution (Beijing Solarbio Science & Technology Co., Ltd., No. G1120) for about 4 min, then performed water washing, differentiation, rinsing back till blue, followed by eosin staining (Beijing Solarbio Science & Technology Co., Ltd., No. G1120) and redyeing for 2 min, then slices were put into 85% alcohol 30 s, subsequently 95% alcohol 30 s, up to 100% alcohol 1 min 100% ii alcohol, to prepare transparent agent and for 3 min each.

### Nissl staining

The slides were rewarmed at 37 °C for 10 min and washed twice with double steaming water for 1 min each time. Then, the tissue was stained with Nissl staining solution (Beyotime, batch number: C0117) for 5 min, and washed with distilled water. This was followed by added into 70% alcohol for 30 s then 100% alcohol for 30 s for dehydration. Lastly, sections were put into transparent agent for 3 min, and sealed in slides.

### Immunofluorescence staining

The eyeball tissue was taken out and rewarmed at 37 °C for 30 min. After washed with PBST for 2 times, at 1 min each, 0.3%Triton + 5% sheep serum was incubated with PBS for 30 min at 37 °C. Then 2% sheep serum dilution with primary antibody(Caspase-1, 1:200, abcam, GSDMD, 1:200, abcam; IL-1β, 1:200, abcam; IL-18, 1:200, abcam; NLRP3, 1:200, abcam) was respectively added on each section with 50 μl/each specimen, and incubated overnight at 4 °C, then rinsed with PBST buffer for 5 min × 5 times. Subsequently, sections were incubated with respective secondary antibody (diluted with PBS), at 37 °C for 1 h, and rinsed with PBST buffer for 5 min × 5 times. Then DAPI was performed to re-stain the nucleus. Three sections from each eyeball were obtained, and 3 fields from each section were performed cell count, for eventual statistical analysis.

### Prediction of potential targets of Scutellarin

PubChem (https://pubchem.ncbi.nlm.nih.gov/) chemical information database was performed to retrieve the 2D structure and molecular formula and obtain SDF file format. Then SDF format file was inputted into Swiss Target Prediction database (http://www.swisstargetprediction.ch/) limited species for "Homo sapiens" to predict its related targets. At the same time, SCU-related targets were predicted by using Genecards database (https://www.genecards.org/) and Traditional Chinese Medicine Systems Pharmacology Database and Analysis Platform (TCMSP, http://tcmspw.com/tcmsp.php). The intersection of the above databases and the deletion of repeated targets are considered as drug targets.

### Screening of targets associated with diabetic retinopathy

Using "Diabetic retinopathy" as a search term, we went into GeneCards database (https://www.genecards.org/) and OMIM database (https://omim.org/) to retrieve related genes of human diseases, to obtain relevant target information of DR, and made intersection of two databases. The pyroptosis-related genes were screened form GeneCards database. Eventually, we analyzed online through the Draw Venn Diagram website, Venn diagram of SCU-DR-pyrophosis targets were drawn and its intersection target was obtained.

### Analysis of gene function and pathway enrichment

The above intersection targets were imported into DAVID website (https://david.Ncifcrf.gov/), using "Functional Annotation Tool" and screening with P < 0.05 as the standard to conduct GO enrichment analysis. The analysis included Biological Process (BP), Molecular Function (MF) and Cellular Component (CC), and the top 10 were selected according to the order of P value. At the same time, KEGG (Kyoto Encyclopedia of Genes and Genomes) pathway analysis was performed on the intersection targets, and the top 20 Genomes were selected according to P < 0.01 as the screening criterion. Lastly, the website of KEGG pathway was referred from www.kegg.jp/feedback/copyright.html^10,11^.The data were acquired using the bioinformatics platform (http://www.bioinformatics.com.cn) for visual processing.

### Construction of protein–protein interaction (PPI) network

The predicted genes were inputted into STRING database (https://string-db.org/) for retrieval, and the protein species was set as "Homo sapiens" with the minimum interaction threshold of 0.9.

### Molecular docking

Scutellarin was imported into PubChem database (https://pubchem.ncbi.nlm.nih.gov), to obtain 2D structure; while the key differential proteins were imported into the PDB database (https://www.rcsb.org), to select the appropriate protein structure. The target protein structure was dewatered by PyMOL 2.5.4 software (https://pymol.org/2/)^12^ to separate ligands and receptors. After taken the target protein as the receptor and scutellarin as the ligand, we determined the active site of molecular docking according to the coordinates of the ligand in the target protein complex and set the grid box coordinates and size according to the active pocket of the target protein, then we performed autodock Vina for molecular docking, and select the binding conformation with the lowest free binding energy. Lastly, we used PyMOL software for visual processing.

### Quantitative PCR validation

Retina tissues from Control group, DM group, SCU treatment group were harvested, respectively. Total RNA was extracted by precipitation and drying. The expression of STAT3, AKT1, MAPK14, HSP90AA1, CASP3, DNMT1, EGFR, TNF, CASP1, DNMT3B and PTGS2 genes were detected by quantitative reverse transcription polymerase chain reaction (qRT-PCR). The qRT-PCR amplification protocols consisted of 95 °C for 30 s and up to 40 cycles of 95 °C for 5 s and 60 °C for 34 s according to the manufacturer’s instructions. All primer for each gene were listed as following. GAPDH was designated as internal reference gene (Table 1).

**Table 1:** Real-time polymerase chain reaction primers.

Gene	Sense primer	Anti-sense primer
STAT3	GGCATCAATCCTGTGGTATA	CCAATCGGAGGCTTAGTG
AKT1	CATGAACGAGTTTGAGTACCT	CTCCTTCTTGAGGATCTTCAT
MAPK14	GTTTCCTGGTACAGACCAT	CAGGGGATTGGCACCAAT
HSP90AA1	TAAACTGGACTCGGGGAA	TTTGGTGCCTGACTTGG
CASP3	AGCGTAAGGAAAGGAGAGG	TGGACATCATCCACACAGA
DNMT1	GGAGGCAGATGAAGACGA	GAGCCAGGAGATGCGAT
EGFR	GTAGTGGTGGCCCTTGG	GCTGGGTGTGAGAGGTTC
TNF	CCACCACGCTCTTCTGTC	GCTACGGGCTTGTCACTC
CASP1 DNMT3B PTGS2 GAPDH	CCCTCAAGTTTTGCCCTT AGAGCCGAGAACGGATG ACTCTATCACTGGCATCCG CTCACTGGCATGGCCTTCCGT	CATCAGCTCCGACTCTCC GAGCCCACCCTCAAAGA GAGCAAGTCCGTGTTCAAG GCCTGCTTCACCACCTTCTT

### Statistical analysis

The staining data were analyzed by ImageJ (400 times), and all the data were statistically analyzed by SPSS 21.0 software. One-way analysis of variance was used for comparison among multiple groups, and the following options were selected successively: analysis—comparison mean-one-way analysis of variance—comparison selection (polynomial)—post comparison selection (LSD, Temhane's T2)—options (description, homogeneity of variance, mean graph). According to the analysis results, P < 0.05 was statistically significant. PCR was analyzed with mean and standard deviation and mapped.

### Ethical approval and consent for participation

All procedures were performed in accordance with the guidelines and approval of the Ethics Committee of the Kunming Medical University, numbered with KMMU20220894, all experiments complied with the ARRIVE guidelines.

### Human and animal ethics

No human studies are involved. The animal ethics code is KMMU20220894.

### Public consent

I declare that all authors agree to publish.

## Results

### Morphological Findings

**Figure 1:**
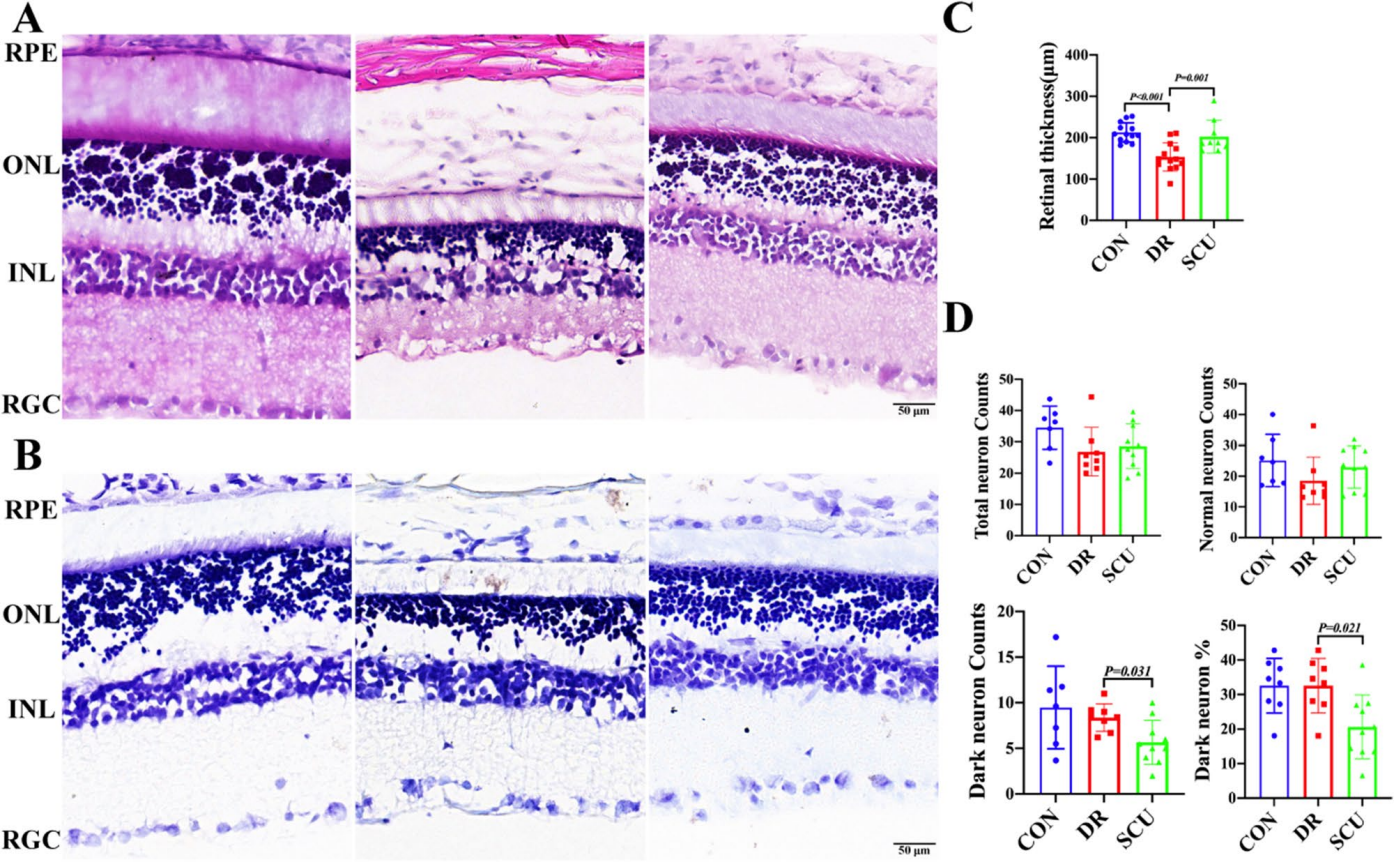
HE staining and Nissl Staining of retinal tissue in rats with diabetic retinopathy treated by SCU. (**A**) HE staining: Control group, diabetic retinopathy group, and SCU treatment group. Bar = 50 μm, *RPE* retinal pigment epithelial cells, *ONL* outer nuclear layer, *INL* inner core layer, *RGC* retinal ganglion cells. (**B**) Nissl staining: Control group, diabetic retinopathy group, and SCU treatment group. Bar = 50 μm, *RPE* retinal pigment epithelial cells, *ONL* outer nuclear layer, *INL* inner core layer, *RGC* retinal ganglion cells. (**C**) Comparison of retinal thickness between Control group, diabetic retinopathy group and SCU group. (**D**) Total retinal neurons, apoptotic neurons, percentage of apoptotic neurons and normal neurons were quantified in Control group, diabetic retinopathy group and SCU group.

In the sham one, all retinal layers were intact and orderly, and the cell morphology was normal. The ganglion cells line regularly with single-layer; the inner core layer was composed of 3–5 cells, and the outer nuclear layer was thick. Comparatively, the retinal cells in DR group were disordered, the ganglion cell layer and the inner and outer nuclear layer were vacuolated, and the retinal structure was thin, which is corresponding to the OCT findings (data are not shown). Moreover, SCU treatment group had clear retinal tissue structure, orderly arrangement of inner and outer nuclear layer cells, with a small amount of vacuolar degeneration. After thickness quantification, it was found that the retinal thinning in the DR group was obvious, while the thinning in the SCU group was significantly reduced (Fig. 1A,C) (P = 0.001).

Nissl staining showed a similar change to HE staining, in which, retina in the Control group rats had clear structure, with orderly and closely arrangement of cells in each layer, and more ganglion cells were characterized by regular morphology. In DR group, the cells of retinal layers were disordered, and there were fewer ganglion cells, irregular shape, and more glial cells. The vacuolar degeneration of ganglion cell layer and inner and outer nuclear layer was obvious. In SCU treatment group, the retinal layers were arranged in order, and the number of ganglion cells increased, compared with DR group. By quantifying the number of ganglion cells, it could be observed that the number of ganglion cells in the Control group was higher than that in the DR group, and the number of ganglion cells increased after SCU treatment, compared with DR group. Moreover, Quantitative statistics showed that the number of pyroptosis neurons decreased significantly after SCU treatment (P = 0.031), and the percentage of pyroptosis neurons was low after SCU treatment (P = 0.021) (Fig. 1B,D).

### Immunofluorescence

**Figure 2:**
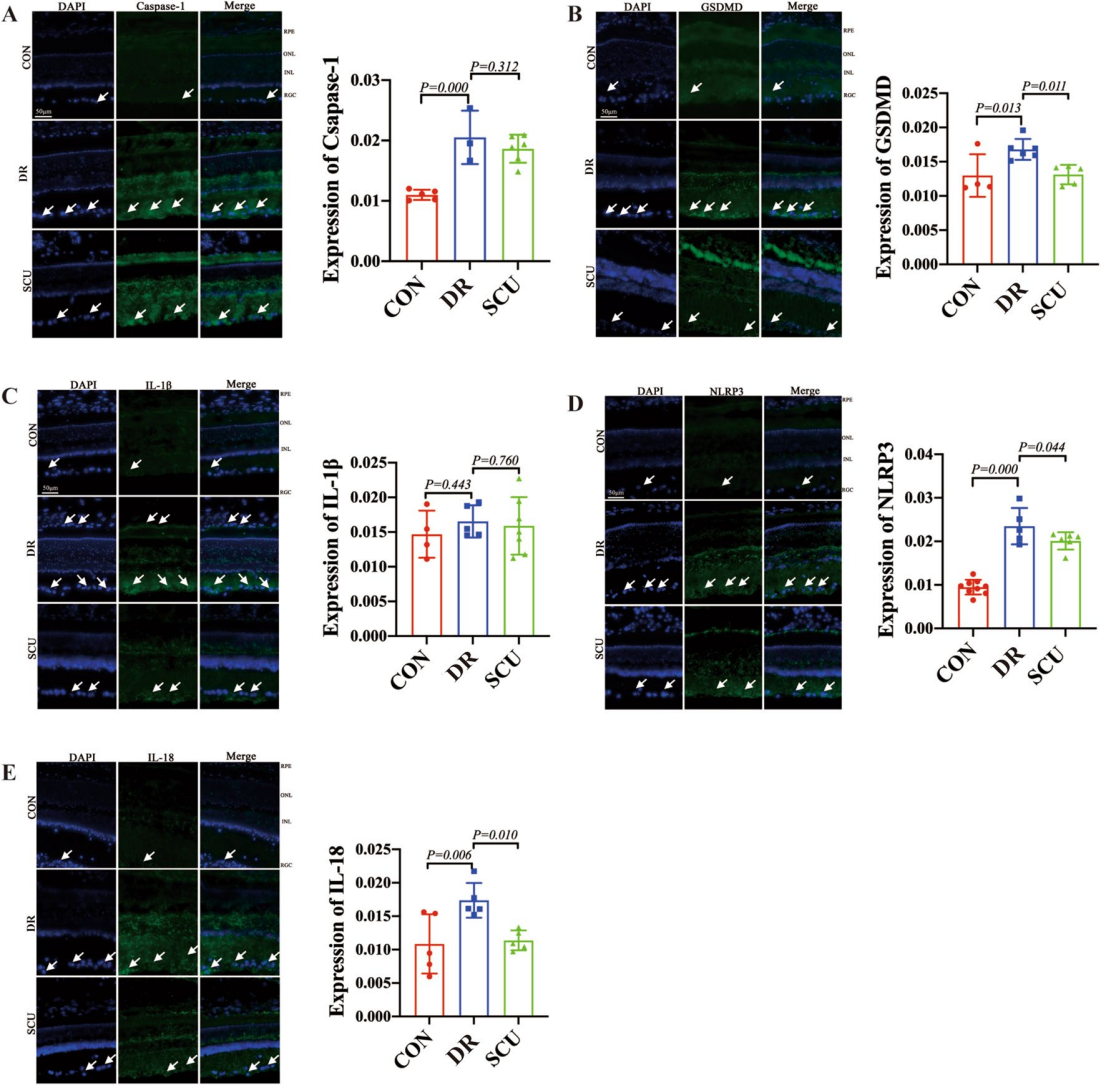
Mean optical density of Caspase-1, GSDMD, IL-1β, NLRP3 and IL-18 in retinal tissues of diabetic retinopathy rats treated with SCU. (**A**–**E**) Control group, DR group and SCU treatment group: Immunofluorescence staining of Caspase-1, GSDMD, IL-1β, NLRP3 and IL-18. Control group, DR group and SCU treatment group: Comparison of the average optical density of caspase-1, GSDMD, IL-1β, NLRP3 and IL-18 immunofluorescence staining.

The results of immunofluorescence staining showed that Caspase-1 was mainly distributed in the lamellar nucleus and retinal ganglion cells (RGC) of the rat retina and located in the cytoplasm. Quantitative analysis of fluorescence intensity statistically showed that compared with the normal group, the average optical density of Caspase-1 in the DR group was significantly increased, and the average optical density of Caspase-1 in the SCU group showed a significant decrease compared with DR group (Fig. 2A). GSDMD was mainly distributed in the inner and outer nuclear lamina and retinal ganglion cells of rats and located in the cytoplasm. Compared with the DR group, the average optical density of GSDMD in the SCU treatment group showed a decreasing trend (P < 0.05) (Fig. 2B). Positive cells for IL-1β were mainly distributed in the cytoplasm of rat retinal pigment epithelial cells (RPE), inner and outer nuclear layers, and retinal ganglion cells, and there were more IL-1β positive cells in DR group that seen in sham group. Quantitative analysis of fluorescence intensity statistically showed that the average optical density of the DR was higher in DR than that of the normal group, and have significant difference between the SCU treatment group and the diabetic group (Fig. 2C). INLRP3 positive cells were mainly distributed in the lamina and ganglion cells of the rat retina and located in the cytoplasm. Quantitative analysis showed that the average optical density of DR was significantly higher than that of the normal group (P < 0.001), and there was significant difference in the average optical density between the SCU treatment group and the DR group (Fig. 2D). IL-18 positive cells were mainly distributed in the inner and outer nuclear layers and retinal ganglion cell layers of rats, and the cells were in the cytoplasm. There were more IL-18 positive cells in DR group, and the average optical density of IL-18 was higher than that of the normal group, while it gets low in SCU group (Fig. 2E).

### Selection on the intersection target of SCU in DR

**Figure 3:**
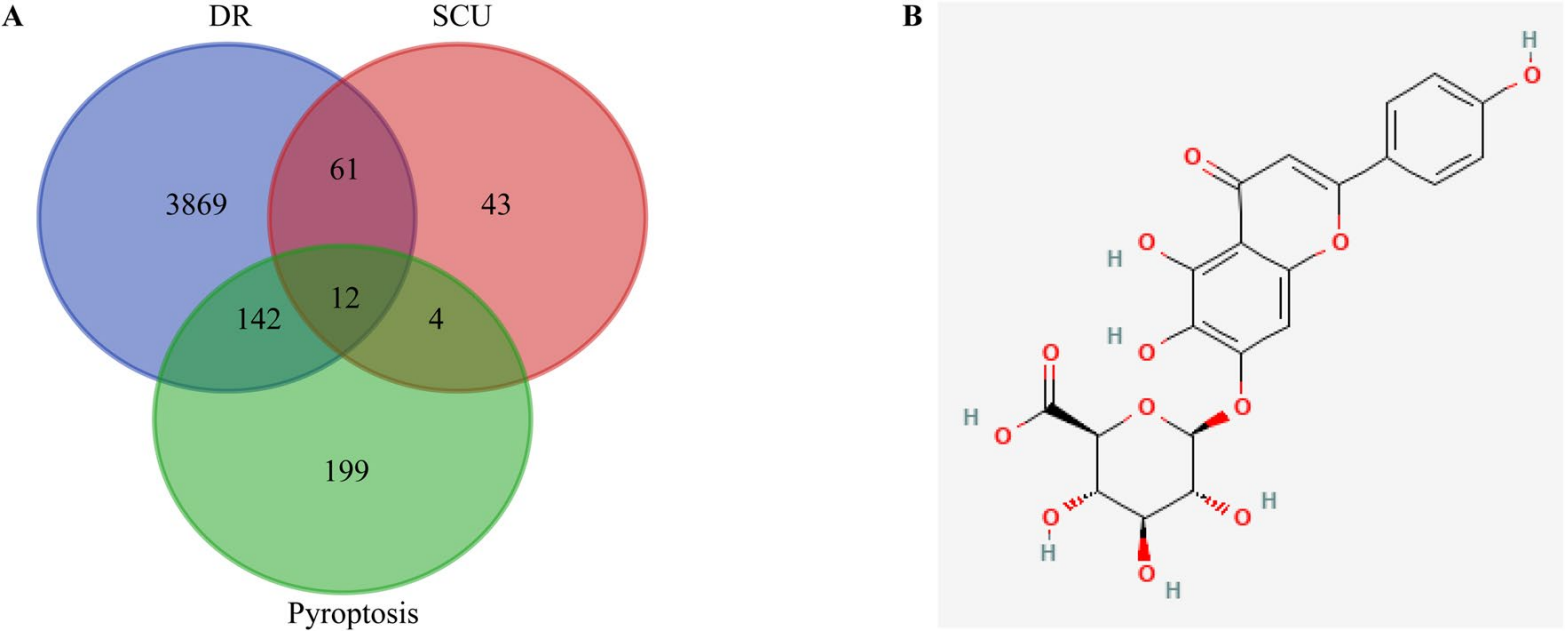
Venn diagram and 2D structure of SCU. (**A**) Venn diagram of the targets of SCU for the treatment of DR. (**B**) 2D structure of SCU.

Six components were obtained by inputting the drug monomer "scutellarin" into the TCMSP database; 100 targets were obtained by inputting the 2D structure of SCU into the Swiss Target Prediction database; 22 targets were gained by using GeneCards database and 120 targets were screened from the three databases. Moreover, 4084 DR related targets were received using GeneCards and OMIM databases; and 357 targets of pyroptosis were obtained from GeneCards database. Finally, 12 intersection targets were screened using online drawing to make a venn diagram (Fig. 3).

### GO results

**Figure 4:**
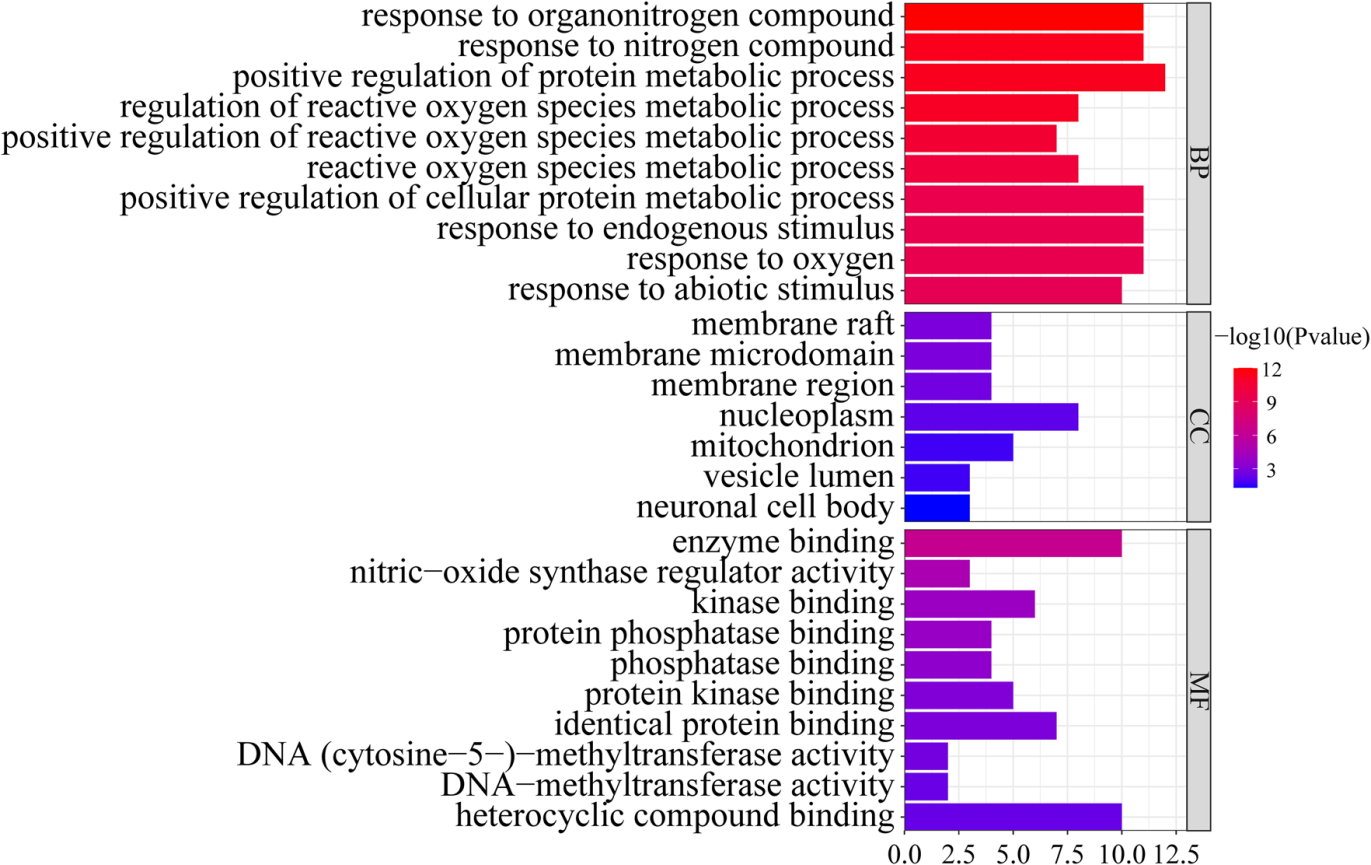
GO enrichment analysis.

GO (Gene Ontology) is a database established by the Gene Ontology Consortium, which aims to establish a semantic vocabulary standard for defining and describing the functions of genes and can be updated with further research and applied to all species. By establishing a dynamic controlled vocabulary to describe the roles of genes and proteins in cells, the properties of genes and gene products in organisms can be comprehensively described. GO enrichment analysis was conducted on 12 intersection targets through DAVID website, including BP, MF, CC and other three modules. With P < 0.05 as the standard, 659 BP related items, 7 CC related items, and 30 MF related items were enriched. Of these, the top 10 GO items of BP, CC and MF were selected according to P values (Fig. 4).

### KEGG pathway detection

**Figure 5:**
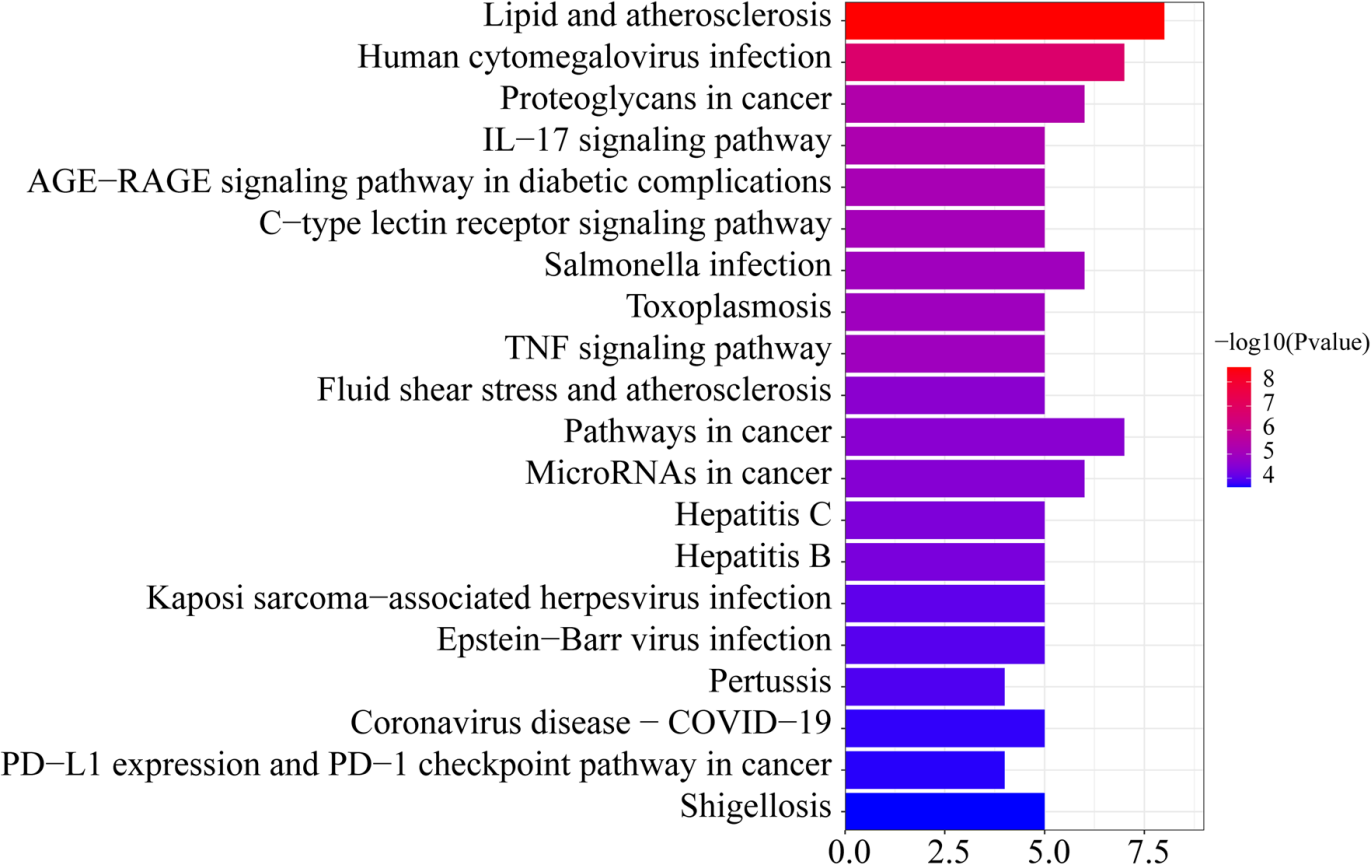
KEGG enrichment analysis.

KEGG enrichment analysis was conducted on 12 intersection target genes through DAVID website. As a result, a total of 70 signal pathways were enriched with P < 0.01 as the standard. The first 20 were selected as the main signal pathways according to the size of P value (Fig. 5). When the length becomes longer, the more genes are enriched in this pathway; and the color with the darker indicated that the P value is the smaller.

KEGG pathway was referred to www.kegg.jp/feedback/copyright.html. and passed the Kanehisa laboratory have happily provided permission^10,11^.

### PPI network construction and core target screening selection

**Figure 6:**
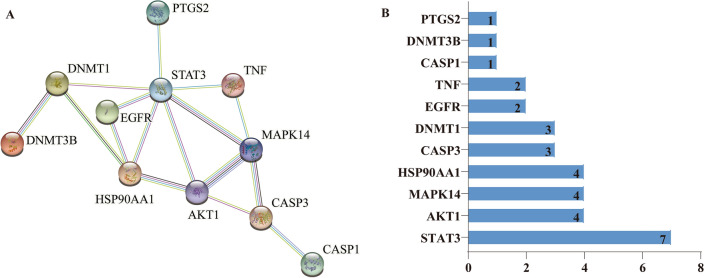
Protein–protein interaction Network. (**A**) PPI network. (**B**) The degree value of the targets.

The 12 intersection targets were imported into STRING database to obtain PPI network diagram (Fig. 6A) and related data files, then they were imported into Cytoscape3.8.0 for intuitive topological analysis. Lastly,CytoHubba was used to calculate the degree value of the targets, and the screening results were sorted from large to small respectively: STAT3, AKT1, MAPK14, HSP90AA1, CASP3, DNMT1, EGFR, TNF, CASP1, DNMT3B, PTGS2 (Fig. 6B).

### Findings from molecular docking

**Figure 7:**
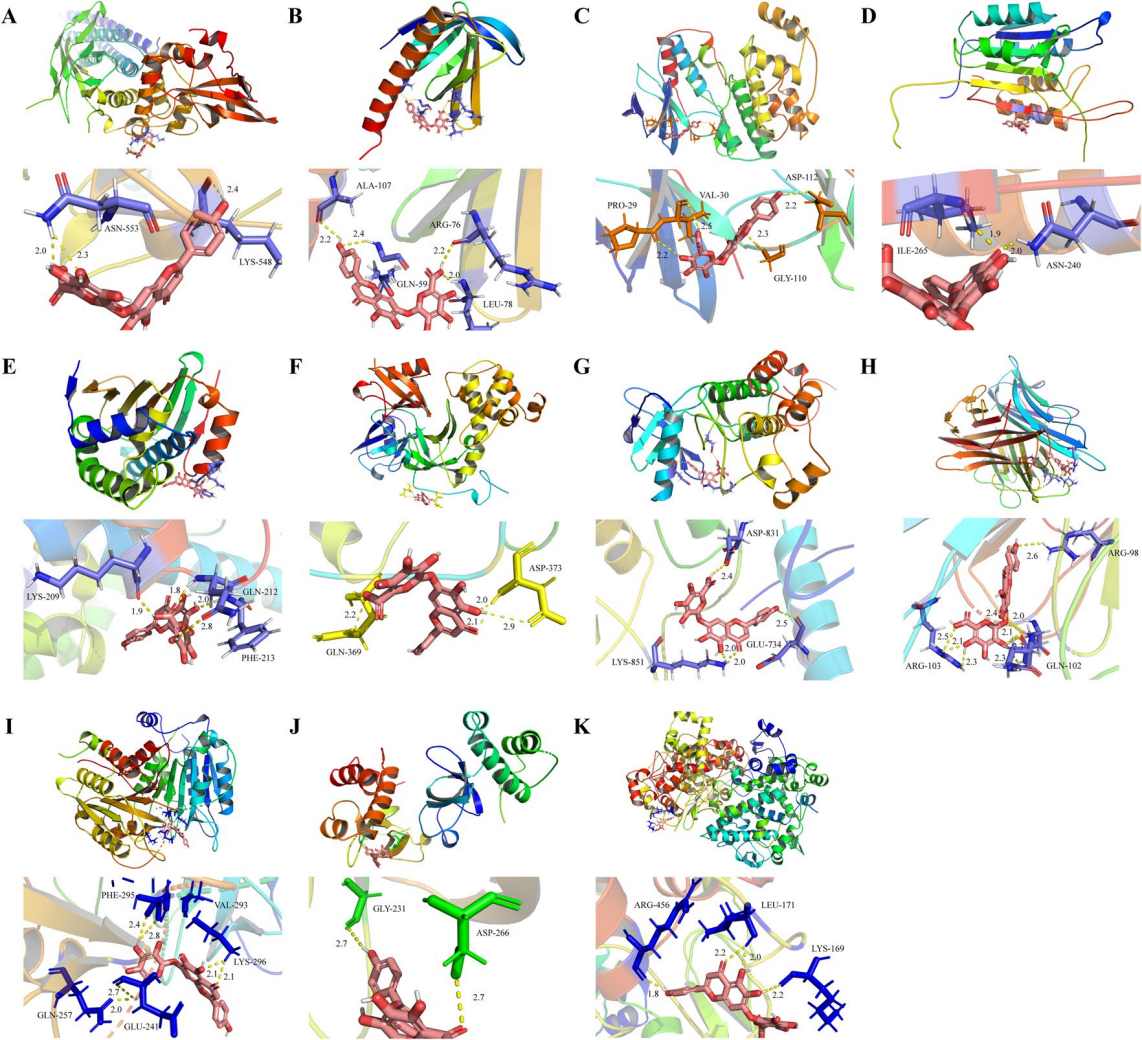
Molecular docking of the hub targets with SCU. (**A**) Binding poses of SCU with STAT3; (**B**) Binding poses of SCU with AKT1; (**C**) Binding poses of SCU with MAPK14; (**D**) Binding poses of SCU with CASP3; (**E**) Binding poses of SCU with HSP90AA1; (**F**) Binding poses of SCU with DNMT1; (**G**) Binding poses of SCU with EGFR; (**H**) Binding poses of SCU with TNF; (**I**) Binding poses of SCU with CASP1; (**J**) Binding poses of SCU with DNMT3B; (**K**) Binding poses of SCU with PTGS2.

The molecular docking between SCU and the core target showed next findings: STAT3 (PDBID:6njs);AKT1(PDBID:1unr);MAPK14(PDBID:1wbs);HSP90AA1(PDBID:4bqg);CASP3(PDBID:3kjf);DNMT1(PDBID:5ydr);EGFR(PDBID:1m14);TNF(PDBID:2e7a);CASP1(PDBID:3e4c);DNMT3B(PDBID:5nv2);PTGS2(PDBID:5f19) were carried out, and the binding energy between the core compound and the core target was calculated to predict their binding activity (Fig. 7). The binding energy lower than 0 indicates that the two molecules can spontaneously bind. The higher corresponding the negative value of binding energy indicates the more stable the concept. As a result, the binding energy of SCU and core targets were − 3.6, − 4.29, − 3.63, − 3.43, − 1.65, − 3.65, − 2.93, − 3.57, − 3.11, − 4.65, − 4.17, respectively.

### The relationship between PPI Hub gene and GO, KEGG pathway

**Figure 8:**
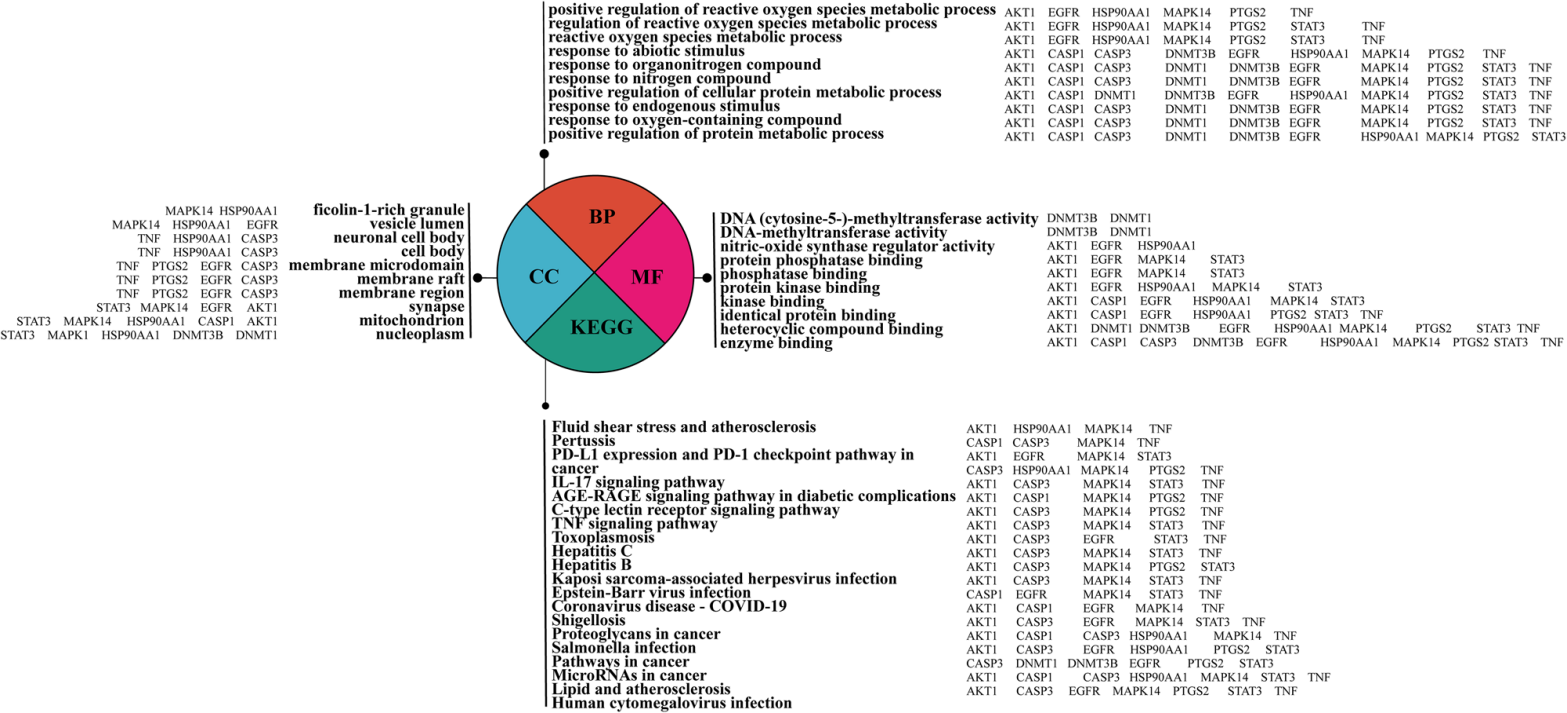
Of relationship between Hub gene and GO and KEGG.

In order to explain the relationship between Hub gene, GO and KEGG, and understand the enrichment of Hub gene, it can be known that in the first 10 pathways of BP, almost every pathway is enriched with 11 Hub genes. Nucleoplasm of the fourth pathway in CC is the most abundant with 7 genes, while the first pathway in MF is the enzyme binding pathway with 9 genes, and the first 8 pathways in MF are enriched with EGFR genes. AKT1 is enriched in 16 pathways among the 20 signaling pathways in KEGG (Fig. 8).

### Detection of Hub genes

**Figure 9:**
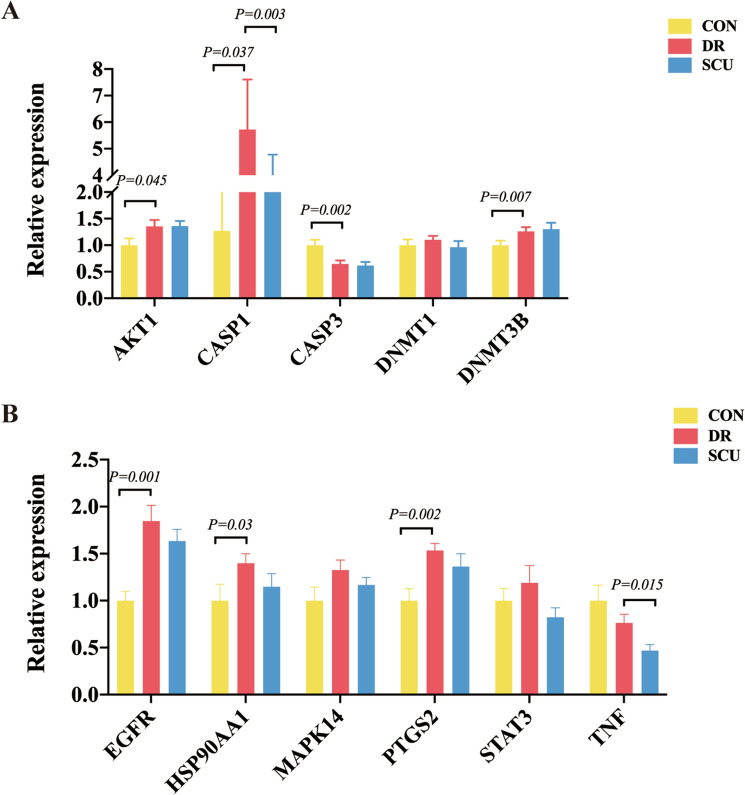
qRT-PCR. (**A**) The relative mRNA expression of AKT1, CASP1, CASP3, DNMT1 and DNMT3B in the control, DR and SCU group. (**B**) The relative mRNA expression of ERFG, HSP90AA1, MAPK14, PTGS2, STAT3 and TNF in the control, DR and SCU group.

We used qRT-PCR to evaluate the mRNA expression of AKT1, CASP1, CASP3, DNMT1, DNMT3B, EGFR, HSP90AA1, MAPK14, PTGS2, STAT3 and TNF in DR and SCU treatment rats. The results showed that the mRNA expression levels of AKT1, DNMT3B, EGFR, HSP90AA1 and PTGS2 were higher in the DR group than in the control group, while, the mRNA expression level of CASP3 was lower in the DR group than in the control group. However, after the treatment of SCU, only the mRNA expression of CASP1 and TNF were decreased compared with the DR group (Fig. 9A,B), which confirmed that CASP1could be considered as a candidate mechanism in SCU treatment DR condition.

## Discussion

In this study, we established a DM model to induce DR, confirmed by HE staining, Nissl staining and immunofluorescence staining. Importantly, the retinopathy of rats in SCU group has been well improved, which confirmed the effect of SCU for the treatment of DR. Moreover, we explained the involved molecular network mechanism. We screened 4084 genes related to DR from GeneCards and OMIM databases. Using GeneCards^13^, TCMSP and Swiss Target Prediction databases^14^, 120 therapeutic targets in SCU treatment group were obtained. Moreover, using GenenCards database, 357 targets related to pyroptosis were retrieved. Subsequently, Drug, disease and phenotypic targets were analyzed online by the Draw Venn Diagram website, and 12 cross targets were obtained. Through the GO function and KEGG pathway enrichment analysis of common targets, 659 BP related items, 7 CC related items, 30 MF related items, and 70 signal pathways were screened out. Lastly, eleven proteins were screened to interact with the cross-target PPI network, and 11 proteins were subsequently docked with the SCU^15^. Lastly, PCR confirmed that SCU can inhibit the pyroptosis reaction of DR through regulating multi-target and multi-pathway system, and most possible mechanism is involved caspase-1, especially in RGC.

### Implication of morphological improvement

Studies showed that the early pathological changes of DR mainly included loss of capillary pericytes, thickening of basement membrane and micro-hemangioma^16^. With the prolongation of the course of DR, the function of vascular and retinal barrier was damaged, capillary occlusion was accompanied by the increase of neovascularization and fibrous tissue formation, and the damage of retinal structure continued to worsen ^17^. In this study, HE staining showed that the retinal cells of the diabetic group were disordered, the ganglion cell layer and the inner and outer nuclear layer had obvious vacuolar degeneration, and the retinal structure becomes thin. Whereas SCU treatment results in clear retinal tissue structure, orderly arrangement of inner and outer nuclear layer cells, and a small amount of vacuolar degeneration. With thickness quantification, it could find that retinal thinning was obvious in the diabetes retinopathy, while the thinning trend was reduced in the SCU treatment group, these suggested that cell damage was improved after SCU treatment in this experiment.

Nissl Staining showed that more ganglion cells exist in the control group and they decreased in the DR group. After SCU treatment, total number of ganglion cells increased, while the number of apoptotic neurons decreased significantly after SCU treatment. It was therefore kept more intact neurons in SCU treatment. In addition, after DR occurred, the thickness of retinal nerve fiber layer, ganglion cell layer and inner plexus layer become thinner and thinner^18,19^. Meanwhile, changes in the number of ganglion cells are marked cells in retinopathy. In our observation, SCU can improve the arrangement of cells and increase the number of ganglion cells to improve the damage of DR, which supported the role of SCU is important in improving DR.

### Immunofluorescent indication

Previously, it has been found that the target cells of some monomer Chinese medicine like SCU were mostly aim to macrophages and endothelial cells. Meanwhile, the main molecular markers involved inflammasome NLRP3, interleukin (IL) 1β and IL-18^7^, protein caspase-1, etc., has been well documented in cell pyroptosis. Caspase-1 can be activated by binding to inflammasome, which processes pro-IL-1β and pro-IL-18 and other inflammatory factors to mature and release and plays a core regulatory role in inflammatory response. In our study, the mean optical density of caspase-1 in the diabetic group was significantly increased compared with the normal group, and it showed a trend of decrease in RGC in the SCU group, compared with the diabetic group^20^, which suggests that SCU can reduce the inflammatory response of DR, especially in RGC. Meanwhile, GSDMD was known to highly express in macrophages, monocytes, neutrophils and CDT8 cells. In this observation, quantitative analysis of fluorescence intensity showed that compared with the diabetes group, the average optical density of GSDMD in the SCU treatment group tended to decrease, supporting caspase-1 regulating GSDMD pathway has been involved in our observation. In addition, NLRP3 is an important signal molecule for tissue cells to regulate inflammatory response, which can promote the expression of downstream caspase-1 protein, activate the pyroptotic pathway, induce inflammatory necrosis, namely cell pyroptotic, and play a key regulatory role in the occurrence and development of DR. Previously, down-regulating the NLRP3/Caspase-1 pathway expression can significantly inhibit the inflammation of retinal tissue induced by high glucose, enhance its antioxidant stress activity, reduce peroxide damage and improve DR symptoms. Meanwhile, the expression of NLRP3, ASC, Caspase-1 and its downstream mature molecules IL-1β and IL-18 were increased in the retina of diabetic rats^21^. In this study, we found that NLRP3 positive cells were mainly distributed in the lamina and ganglion cells of the rat retina, and the cells were located in the cytoplasm, however, only average optical density of the diabetic group was significantly higher than that of the normal group, but there was significant difference between the SCU treatment group and the diabetic group, indicating NLRP3 is involved in the process of SCU treatment. As to IL-1β, it is similar to NLRP3. IL-18, a new and multifunctional proinflammatory cytokine, by enhancing the expression of Fas ligand in Th1 cells and NK cells to mediate cytotoxicity and induce the gene expression and synthesis of IL-1 and TNF-α through IL-18 receptor complex, has been well involved in our experiment^20^, in which, IL-18 positive cells were mainly distributed in the inner and outer nuclear layers and retinal ganglion cell layers of rats. Quantitative analysis of the average optical density showed that the level of IL-18 in diabetic group was higher than that of the normal group, and the average optical density of the SCU group was lower, which suggest that SCU reducing the inflammatory response in DR is involving in IL-18 diabetic retinopathy induced by DM^22^.

### Intersection genes of SCU and the inhibition of pyroptosis in DR

In this study, 12 intersection targets were screened using online drawing to make a venn diagram. According to the above screening results, it can be known that SCU may treat DR through multiple targets.

Pyroptosis is one of the research hotspots in the past decade, and the research progresses rapidly in various systems, bringing new treatment ideas for a variety of refractory diseases. Recent studies have found that^23^ NLRP3 inflammasome is activated in proliferative diabetic retinopathy, and the activation of NLRP3 is a key step in pyroptosis^24^. Moreover, studies found that^25^ SCU attenuated diabetes-induced human retinal endothelial cells (HRECs) proliferation, migration, and tube formation and decreased neovascularization and resistive index in the retina of streptozotocin-induced diabetic rats through oral administration. Mei et al. found that^26^ SCU could protect from blood-retinal barrier damage by focusing on inhibiting microglia-initiated inflammation and subsequent oxidative stress injury. Wang et al. also found that^27^ SCU exhibits an antiangiogenic effect through inhibition of oxidative stress, and enhances HIF-1α degradation and reduction of VEGF secretion. These evidences consolidate that SCU has anti-oxidative stress, anti-angiogenic and anti-inflammatory effects and may be a potential therapy for DR. Meanwhile, it was found that SCU dose-dependently inhibited lipopolysaccharide-induced release of caspase-11 p26 and the production of GSDMD-NT in macrophages, resulting in decreased pyroptosis^28^. In addition, SCU may inhibit ATP-induced NLRP3 inflammasome activation and pyroptosis by regulating the activity of protein kinase A (PKA)^29^, which supported that SCU has the effect of inhibiting pyroptosis in other system. Comparatively, this study is the first to investigate the inhibitory effect of SCUs on DR Scorch death and its related mechanism. Importantly, we reported the intersection gene between SCU and pyroptosis may underly crucial network in SCU improving DR. These findings provide vital evidence for the treatment of DR, administrated by SCU.

### GO enrichment analysis

In this study, GO enrichment analysis was conducted on 12 intersection targets through DAVID website, including BP, MF, CC and other three modules. With P < 0.05 as the standard, 659 BP related items, 7 CC related items, and 30 MF related items were enriched. The top 10 biological processes were response to organonitrogen compound, response to nitrogen compound, positive regulation of protein metabolic process, regulation of reactive oxygen species metabolic process, positive regulation of reactive oxygen species metabolic process, reactive oxygen species metabolic process, positive regulation of cellular protein metabolic process, response to endogenous stimulus, response to oxygen-containing compound. These results indicated that the targets of SCU treatment of DR was mainly involved in the process of reactive oxygen species (ROS) metabolism.

Experiments found that ROS was significantly increased after high glucose-induced bovine retinal endothelial cells and retinal tissue of diabetic mice^30,31^. Similarly, measurements of the vitreous of DR patients also found significantly elevated levels of markers of oxidative stress^32^. The study also found that the activities of antioxidant stress system enzymes including superoxide dismutase, glutathione peroxidase, and catalase were weakened in mouse diabetic retinal tissue^33^. High glucose-induced ROS in retinal tissue can induce apoptosis of retinal ganglion cells, retinal vascular endothelial cells and pericytes^34^. The application of anti-oxidative stress drugs can inhibit the progression of DR^35^. In addition, a large amount of ROS activates NLRP3, which could activate caspase-1, causing pyroptosis. These evidences suggest that these molecules regulate diabetic retinal cell pyroptosis by participating in the process of ROS metabolism.

### KEGG enrichment analysis

In this study, there were 70 signal pathways enriched with P < 0.01 as the standard via DAVID database. Among them, the most important signaling pathways were IL-17 signaling pathway and AGE-RAGE signaling pathway, which suggested that these two pathways played an important role in SCU's inhibition of diabetic retinal pyroptosis.

The study found that the expression and production of IL-17A in DR were increased^36^; high glucose could induce the expression and secretion of IL-17A in retinal Muller cells^37^. Moreover, IL-17A can damage the BRB through the activating the JAK1 signaling pathway^38^. These findings suggest that the proinflammatory cytokine IL-17A is involved in the development and progression of DR lesions.

The AGE-RAGE signaling pathway is a central link in the pathogenesis of DR. Excessive glucose in blood binds with circulating macromolecules (such as fat and histone proteins, etc.) through non-enzymatic pathways to generate stable and irreversible AGEs that are widely accumulated inside and outside cells, leading to pericellular apoptosis and basement membrane thickening, which is an important cause of DR^39^. The increase of AGEs can significantly up-regulate the expression of AGE receptor for advanced Glycation end products (RAGE), and the combination of AGEs and RAGE can induce the increase of reactive oxygen species and inhibit the activity of GAPDH^40^. It can also induce the expression of VEGF and other pro-angiogenic genes, which accelerate pericyte apoptosis and neovascularization growth^41^. In addition, the hypoxic state of the omentum caused by hyperglycemia activates the HIF-1 signaling pathway, also stimulates the expression of VEGF and induces the growth of DR neovascularization^40^. Inhibition of pericellular apoptosis and neovascularization mediated by age-rage signaling pathway is an important approach for the prevention and treatment of DR, which is consistent with the network pharmacological prediction results of SCU related pathways for the prevention and treatment of DR.

### Construction of PPI network and molecular docking

In this study, PPI network results showed that STAT3, AKT1, MAPK14, HSP90AA1, CASP3, DNMT1, EGFR, TNF, CASP1, DNMT3B, PTGS2 exited interaction. Among them, STAT3 has the highest degree value, which may be the key target of SCU for DR treatment. Molecular docking results showed that these key target proteins could stably bind to SCU and form hydrogen bonds, which preliminarily revealed the role characteristics of SCU in multi-target and multi-pathway treatment of DR.

Latest research found that^42^ STAT3 activation increased in DR because STAT3 activation is associated with inflammation. STAT3 activation in microglia played an important role in pericyte apoptosis in DR through increased TNF-α expression. Other studies have found that^42^ melatonin deactivated microglia via inhibition of PI3K/Akt/STAT3/NF-κB signaling pathways, thus maintaining the integrity of iBRB. These suggests that STAT3 may be involved in BRB damage. Interestingly, circZNF532 and STAT3 were upregulated but miR-20b-5p was downregulated in the serum samples of patients with DR and HG-induced ARPE-19 cells. Elevated miR-20b-5p or circZNF532 knockdown enhanced proliferation but reduced apoptosis and pyroptosis of ARPE-19 cells^43^, suggesting that STAT3 may be involved in pyroptosis and thus promote the occurrence and development of DR.

Retinal endothelial cell under high-glucose conditions increased levels of Src kinase, phosphatidylinositol 3-kinase/Akt1/endothelial nitric oxide synthase, and ERKs. The sustained activation of these signaling pathways was essential for enhanced migration of retinal EC under high-glucose conditions^44^. In addition, PI3K/AKT/mTOR pathway, has involved in crucial cellular functions such as cell proliferation, migration and angiogenesis, and AKT1 was one of the key target genes of PI3K^44^. Furthermore, el-Remessy AB et al. proposed that^45^ high glucose treatment blocks the pro-survival effect of VEGF and causes accelerated endothelial cell apoptosis via the action of peroxynitrite in causing tyrosine nitration of PI 3-kinase, so as to inhibit activity of Akt-1 kinase and increasing the activity of p38 MAP kinase. In addition, Wang et al. also pointed out that^46^ MAPK14 was identified gene candidate of potentially involved in DR, which was consistent with the results of this study. Saik OV etc. found that^47^ HSP90AA1 occupied central positions in the networks of DR and were associated with response to hypoxia. CASP3 has been involved in apoptosis, which cause DR through retinal damage^48^. What’s more, under high glucose conditions, the viability of retinal pigment epithelial cells was decreased and the apoptosis rate increased, the expression of CASP3, Bax were increased and the expression of Bcl-2 decreased^49^. Meanwhile, a study found that^50^ STZ injection caused the increased expression levels of DNMT1 and DNMT3B, which induced DNA hypermethylation, inhibited the expression of CDKN2B and facilitated the damages of RGC. Especially, DNMT1 could promote MEG3 promoter methylation to inhibit MEG3 expression by recruiting methyltransferase, which activated the PI3K/Akt/mTOR signaling pathway to accelerate endothelial-mesenchymal transition in DR^51^. Zhu et al. discovered that^52^ DNMT1-mediated peroxisome proliferator-activated receptor alpha methylation promotes apoptosis and ROS levels of human retinal capillary pericytes and aggravates damage of retinal tissues in DR of mice, which highlighted novel insights into DR pathogenesis. Dolinko AH etc. pointed out that^53^ DNA methylation genes, such as DNMT3A and DNMT3B, were upregulated in diabetic human RPE cybrids.

For TNF-α, study indicated that the expression of TNF-α mRNA was significantly increased in diabetic rats induced by a single intraperitoneal injection of 60 mg/kg STZ, and Adeno-associated virus (AAV)-mediated expression of human TNF-α in the murine eye induces retinal inflammation^54^. More importantly, TNF-α released from the activated microglia induced BRB damage, which in turn leaded to inflammation of the retinal^55^.

The research concluded that^56^ EGFR critically promoted retinal dysfunction, retinal structural impairment, and retinal vascular abnormalities in models of DR and could be a potential important therapeutic target for treatment of DR. Moreover, in the DR rat model, EGFR/PI3K/AKT pathway was activated and accelerated the invasion of endothelial cells and retinal pericytes^57^. More importantly, clinicopathological information presented that EGFR level was strongly associated with DR pathogenesis. These results pointed that EGFR was a potential target for SCU treatment of DR.

In the research to investigate the effect of VEGFR1 blockade on DR, VEGFR1 blockade interfered with the expression of 10 novel cytokines and chemokines, including CASP1^58^. Importantly, research applied advanced glycation end product modified bovine serum albumin (AGE-BSA) to simulate the DR environment suggested that^59^ AGE-BSA induced the active cleavage of CASP1, indicating the occurrence of CASP1-mediated pyroptosis in human retinal pericytes.

Together, our results demonstrated that SCU may regulate the reactive oxygen species metabolic process by multiple networks including STAT3, AKT1, MAPK14, HSP90AA1, CASP3, DNMT1, EGFR, TNF, CASP1, DNMT3B and PTGS2.

### The relationship between PPI Hub gene and GO machine KEGG pathway

In order to explain the relationship between Hub gene, GO and KEGG, and understand the enrichment of Hub gene, it can be known that in the first 10 pathways of BP, almost every pathway is enriched with 11 Hub genes. Nucleoplasm is the most abundant gene in the fourth channel of CC, with seven genes being enriched. The first pathway of MF is: enzyme binding, enriched with 9 genes, and the first 8 pathways of MF are enriched with EGFR gene. AKT1 was enriched in 16 of the 20 KEGG signaling pathways. Thus, it can be inferred that the relationship between GO analysis and Hub gene is as follows: Hub gene has the most extensive enrichment in the BP pathway, followed by MF gene and CC gene. The relationship between KEGG and Hub genes is as follows: 7 genes are enriched in the first pathway: Lipid and atherosclerosis, and AKT1 is enriched in 16 pathways, which explains that AKT1 has a regulatory effect on KEGG pathway.

### Quantitative PCR findings and its functional implication

In our study, qPCR confirmed in retina of in DR, AKT1, DNMT3B, HPS90, PTG32 exhibited a marked increase, but they didn't exhibit a significant change in SCU treatment group, which indicated that these core molecules are involving in DR injury, however, they are not positive in SCU administrated condition. Comparatively, caspase-1 and EGFR showed a striking presentation, in which, they increased in DR condition, and decreased in SCU treatment rats, which confirmed these two molecules are important in our experimental conduction. As to EGFR, it has been well known that its increase is a reaction of DR, and its inhibition is useful to the improvement of DR. In this study, the most important result is that caspase 1, an activated pyroptosis gene, not only found in immunodeficient staining, but also in qPCR detection. Therefore, it seems caspase-1 is important in SCU treatment condition. We guessed that caspase-1 could be considered as a crucial target with SCU in this experiment.

STAT3, widely expressed in the retina and other tissues, could regulate the cell growth, differentiation and angiogenesis and participate in the pathogenesis of DR^60^. STAT3 was upregulated after the first day, reached the peak after 1 week and downregulated gradually in the following weeks^61^. Similarly, one study^62^ found that the mRNA expression of STAT3 was remarkably high in model group with the activation of NDRG2/IL-6/STAT3 signaling pathway. The evidence suggested that STAT3 was also involved in the occurrence and development of DR. Previous study^44^ showed that inhibit activity of AKT1 resulted in the apoptosis of endothelial cells induced by high glucose. More evidence^63^ pointed that diabetes caused apoptosis of neural and vascular cells in the retina. Cao et al.^64^ found that l-Carnitine, an endogenous mitochondrial membrane compound, inhibited the cell apoptosis induced by high glucose, characterized by the downregulation of CASP3, CASP9 and Bax/Bcl-2. What's more, chronic low-grade inflammation also played an important role in the DR. Some studies found that increased various pro-inflammatory cytokines in the retina and vitreous humor of diabetic patients and animals included TNF-α, NF-kB and IL 1, which were important for the development of DR. Saik etc.^47^ found that HSP90AA1 occupied central positions in the networks of DR and were associated with response to hypoxia. What's more, HSP90AA1 had higher expression in healthy human foveomacular retina. It has been reported^56^ that EGFR promoted retinal dysfunction, retinal structural impairment, and retinal vascular abnormalities in models of DR. What's more, EGFR level was strongly associated with DR pathogenesis. One study^65^ found that STZ injection caused the increased expression levels of DNMT1 and DNMT3B, which induced DNA hypermethylation and facilitated the damages of RGC. Zhu et al.^52^ suggested that DNMT1-mediated peroxisome proliferator-activated receptor alpha methylation aggravated damage of retinal tissues in DR mice. More importantly, Clinical research^4^ showed that DNA methylation genes, such as DNMT3A and DNMT3B, were upregulated in diabetic human RPE cybrids. Accumulating evidence in preclinical studies indicated a key role of MAPK14 in inflammation. The p38 MAPK pathway was also associated with the induction of inflammatory molecules. What's more, MAPK14 inhibitors have been shown to suppress production of IL-6, TNF, and IL-1 in vitro and in vivo in other diseases. Yin et al.^66^ observed CASP1 expression in the RGC and INL by immunohistochemistry, and they found increased CASP1 expression in the retina of diabetic rats. Clinical trials also found that CASP1 in the vitreous of DR patients increased with increasing VEGF levels, especially in PDR patients. These suggested that CASP1 is important in DR and our observation added new consolidate evidence.

## Conclusion

SCU treatment showed a positive effect in inhibiting RGC pyroptosis in DR, and underlying mechanism is involving in multi-target and multi pathway. Of these, caspase-1, IL-1β, IL-18, GSDMD and NLRP3 in RGC could be considered as more important point.

## Data Availability

All data generated or analyzed during this study are included in this published article.
